# Effects of porcelain thickness on the flexural strength and crack propagation in a bilayered zirconia system

**DOI:** 10.1590/1678-7757-2015-0479

**Published:** 2017

**Authors:** Viviane Maria Gonçalves de Figueiredo, Sarina Maciel Braga Pereira, Eduardo Bressiani, Márcia Carneiro Valera, Marco Antônio Bottino, Yu Zhang, Renata Marques de Melo

**Affiliations:** 1 Univ. Estadual Paulista, Instituto de Ciência e Tecnologia de São José dos Campos, Departamento de Materiais Odontológicos e Prótese, São Jose dos Campos, SP, Brasil; 2 Univ. Estadual Paulista, Instituto de Ciência e Tecnologia de São José dos Campos, Departamento de Odontologia Restauradora, São Jose dos Campos, SP, Brasil; 3 New York University, College of Dentistry, Biomaterials and Biomimetics, New York, USA

**Keywords:** Ceramics, Porcelain, Mechanical stress, Confocal microscopy

## Abstract

**Objective:**

This study evaluated the influence of porcelain (VM9, VITA Zahnfabrik, Germany) thickness on the flexural strength and crack propagation in bilayered zirconia systems (YZ, VITA Zahnfabrik, Germany).

**Material and Methods:**

Thirty zirconia bars (20.0x4.0x1.0 mm) and six zirconia blocks (12.0x7.5x1.2 mm) were prepared and veneered with porcelain with different thickness: 1 mm, 2 mm, or 3 mm. The bars of each experimental group (n=10) were subjected to four-point flexural strength testing. In each ceramic block, a Vickers indentation was created under a load of 10 kgf for 10 seconds, for the propagation of cracks.

**Results:**

The results of flexural strength were evaluated by One-way ANOVA and Tukey's test, with a significance level of 5%. The factor “thickness of the porcelain” was statistically significant (p=0.001) and the l-mm group presented the highest values of flexural strength. The cracks were predominant among the bending specimens with 1 and 2 mm of porcelain, and catastrophic failures were found in 50% of 3-mm-thick porcelain. After the indentation of blocks, the most severe defects were observed in blocks with 3-mm-thick porcelain.

**Conclusion:**

The smallest (1 mm) thickness of porcelain on the zirconia infrastructure presented higher values of flexural strength. Better resistance to defect propagation was observed near the porcelain/ zirconia interface for all groups. Higher flexural strength was found for a thinner porcelain layer in a bilayered zirconia system. The damage caused by a Vickers indentation near and far the interface with the zirconia shows that the stress profiles are different.

## Introduction

Ceramic materials are increasingly used in dental restorations because of the excellent combination of esthetic, biological, and mechanical properties, such as wear resistance and rigidity^20^, making them a material of choice for oral rehabilitation^11^ ^,^ ^12^. Dental ceramics can be used for framework, e.g., yttrium-oxide partially stabilized zirconia, which has high flexural strength and fracture toughness, but limited translucency^20^ ^,^ ^26^. Excellent esthetic properties are achieved by applying a veneering ceramic (feldspathic glass or porcelain) to the framework^23^.

The most common clinical complications associated with the infrastructure of zirconia restorations are loss of retention, the need for endodontic treatment, veneering ceramic fractures and bleeding on probing^12^. Mechanical complications such as porcelain fracture tend to be more prevalent in veneered zirconia crowns than in those with metal coping^18^ ^,^ ^24^. Clinical reasons for the failure of these ceramic restorations include mechanical stress for high occlusal loads, which causes crack propagation and later results in “chipping”^20^ ^,^ ^28^ and location in the dental arch^21^. From a biomechanical materials perspective, residual stresses in compression and tension generated along the porcelain layer play a critical role in the failure of porcelain veneered zirconia crowns^27^.

Residual stresses influence the strength and fracture behavior of ceramic crowns and when combined with functional stresses may lead to restoration failure^17^. Among the factors that control the amount of residual stresses are the geometry of the infrastructure^6^ ^,^ ^8^ ^,^ ^16^ ^,^ ^19^, the thickness of the ceramic overlay and framework^16^, and the cooling rate at temperatures above the glass transition temperature^10^ ^,^ ^15^. The nature of stresses, either compressive or tensile, also affects ceramics strength, the former increases strength and the latter increases crack propagation under occlusal loadings.

From a clinical viewpoint, the thickness of zirconia framework may vary from 0.3 to 1.0 mm, while that of the veneering ceramic varies from 0.3 to 3.0 mm^22^ ^,^ ^29^. This variation in thickness is due to the dental crown complex shape, which has regions, such as cusps and axial walls built up with varying porcelain thicknesses, promoting different stress magnitudes^27^. Few studies have specifically addressed the mechanical behavior of different thicknesses of porcelain-zirconia^25^. Therefore, it is necessary to know the effects of mechanics and crack propagation at different thicknesses of porcelain-zirconia infrastructure performance.

The methodologies used to analyze the cracks in ceramics include microtomography^19^, transillumination^3^, and Scanning Electron Microscopy (SEM). However, the literature does not report crack behavior of ceramic bilayer system with regard to its depth, volume, and the extent of dependence on porcelain thickness. This study aimed to evaluate the influence of porcelain-zirconia thickness on the flexural strength and crack propagation of a bilayered system by means of confocal microscopy analysis. The hypotheses to be tested were HI - The smallest thickness of veneering ceramic increases flexural strength; and H2 - There will be differences in the propagation of defects (indicative of residual stresses), depending on veneering ceramic thicknesses.

## Material and Methods

**Figure 1:**
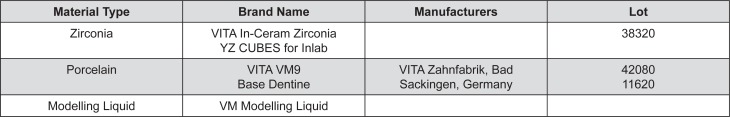
Material type, brand name, and manufacturers used in the study

The materials used in this study are shown in Figure 1.

### Sample preparation

In this study, zirconia blocks pre-synthesized with dimensions 14x15x20 mm (VITA In-Ceram^®^ YZ Cubes, Vita Zahnfabrik, Bad Säckingen, Germany) were used to make bar and block specimens. Thirty bar-shaped zirconia specimens, with dimensions of 20.0 mm length x 4.0 mm width x 1.0 mm thickness, and six block-shaped zirconia specimens, with dimensions of 12.0 mm length x 7.50 width x 1.2 mm thickness, were cut with the aid of a cutting machine (1000 ISOMET, Buehler Ltd., Lake Bluff, IL, USA). Specimen dimensions were obtained after a sintering process in a Zyrcomat furnace (Vita Zahnfabrik, Bad Sackingen, Germany).

Specimens were cleaned in an ultrasonic bath (Vitasonic, Vita Zahnfabrik, Bad Sackingen, Germany) for 5 min in 10% isopropyl alcohol to remove any residue from previous steps and dried at room temperature. They were then randomized into experimental groups, with 10 bars and 2 blocks for each group. The groups followed the porcelain thickness, accordingly:

Group 1: Bars and blocks with 1 mm of zirconia and 1 mm of porcelain.

Group 2: Bars and blocks with 1 mm of zirconia and 2 mm of porcelain.

Group 3: Bars and blocks with 1 mm of zirconia and 3 mm of porcelain.

### Application of veneering ceramic

The application of porcelain (VM9; Vita Zahnfabrik, Bad Sackingen, Germany) was performed manually. The zirconia specimens were inserted into a silicone mold for the application of porcelain layers. Thus, the ceramic powder was mixed with the modeling liquid, applied, and condensed onto zirconia bars. The powder used was Base Dentin (VM9; Vita Zahnfabrik, Bad Sackingen, Germany), which was applied with a brush under vibration.

The firing process was the basic cycle for VITA VM 9 (recommended by the manufacturer). Base Dentin was first applied until completion of the desired thickness and fired in a Vacumat 6000 (Vita Zahnfabrik, Bad Sackingen, Germany) furnace in two firing cycles for all groups (Table 1). Bar and block shaped specimens were then finished with silicon carbide sandpaper #600, 800, 1000, and 1200 (3M, Sumaré, SP, Brazil), under constant water irrigation (Automet, Buehler, Lake Bluff, IL, USA).

**Table 1:** Basic firing cycle for VITA VM 9 according to the manufacturer

Firing process	Starting T (°C)	Pre-drying t (min) and closing t (min)	Heating t (min)	Heating rate (°C/min)	Firing T (°C)	Holding t (min)	Vacuum holding t (min)
Wash Firing	500	2	8.11	55	950	1	8.11
1^st^ dentine Firing	500	6	7.27	55	910	1	7.27
2^nd^ dentine Firing	500	6	7.16	55	900	1	7.16

### Four-point flexural strength test

The strength of the bilayer system was measured by the four-point bending test. Specimens were placed in a four-point bending fixture (5-mm-diameter rollers, supports spaced 16 mm apart, and rollers 8 mm apart), where the porcelain was tested under tensile stress. The flexural strength test was performed at a speed of 0.5 mm/min, with a 1000-kgf load cell, in a universal testing machine, EMIC DL 1000 (São José dos Pinhais, PR, Brazil). Maximum load (P) was recorded at the first sign of fracture, verified by the first cracking sound and changes in the deflection curve. Strength values (a) were calculated for flexural strength of the bilayer according to the following equation^9^:

(1)
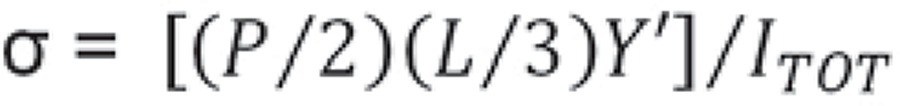


where *P* is the applied load in Newtons (N), *L* is the distance between the support rollers in millimeters (mm), *Y′* is the distance in mm from the neutral axis of the fiber, and outer *I_TOT_* is the moment of inertial cross-section around the central axis.

The *Y′* value was given by equation 2:

(2)
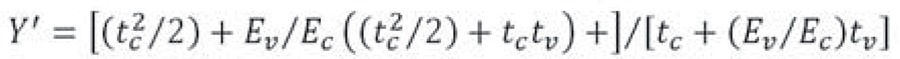


where *t_c_* and *t_v_*, correspond to the thickness (T in mm) of the zirconia infrastructure and porcelain, respectively, and *E_c_* and *E_v_* are the elastic modulus (E) of the ceramic layers of infrastructure and porcelain, respectively.

The *I_TOT_* variable was determined according to equation 3:

(3)
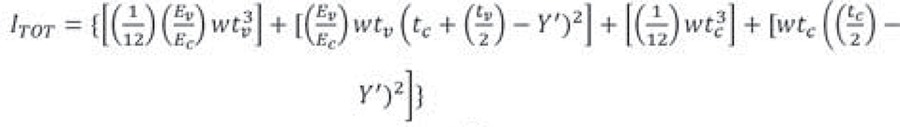


and *w* is the sample width.

Material thicknesses were measured with a digital caliper, and the elastic moduli obtained from the literature were 209.3 GPa for YZ and 66.5 GPa for VM9^7^. The glaze layer was not used in this study and, therefore, the values for this material were not considered.

### Analysis by stereomicroscopy

The failures of the bar-shaped specimens were observed with a stereo microscope (Discovery z-20, Zeiss, Jena, Germany) at lOx and 40x magnification, and classified according to Lima, et al.^15^ (2013) as: cracking, a fissure/crack in the porcelain layer; delamination/chipping of veneering ceramics; total removal of porcelain from the zirconia layer, leaving YZ free of veneering ceramics; or catastrophic failure.

### Vickers indentation

A defect in the six block-shaped specimens was simulated by a Vickers indentation, performed with a load of 10 kg for 10 s. Prior to that, the blocks were embedded in acrylic resin and wet-ground with SiC sandpaper in the same way the bending specimens were.

**Figure 2:**
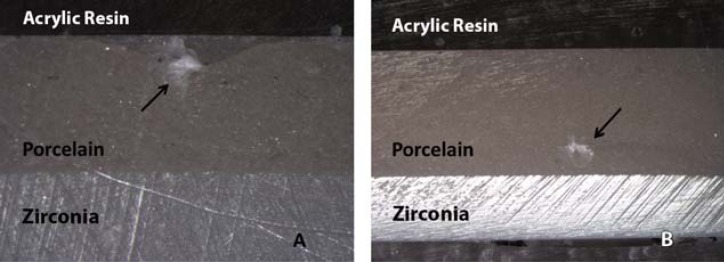
Vickers indentation. Far (A) and close to the interface (B) (40x)

Each block received two indentations (Figure 2), either close to (0.3 mm from the interface) or far from (0.3 mm from the upper surface of the porcelain) zirconia. Vickers indentation was performed using a DHV-1000 Micro Hardness Tester (Durometer MD-1; Microtest, Asker, Kyoto, Japan). As a high loading force would lead to interactions between the cracks and impair the evaluation, we performed only one indentation on each site.

### Confocal microscopy analysis

Damage sustained from the Vickers indentation were examined in a 3D confocal microscope (Mitutoyo, Suzano, SP, Brazil), whereby the depth, extension, and volume of the defect obtained were measured using the appropriate software (Cyber CT Scan 8, Ingolstadt, Germany).

### Statistical analysis

Results of flexural strength were analyzed by One-way ANOVA at a significance level of 5%. Tukey's test identified differences among groups. Results of crack propagation analysis were evaluated by measures of central tendency and standard deviations.

## Results

**Figure 3:**
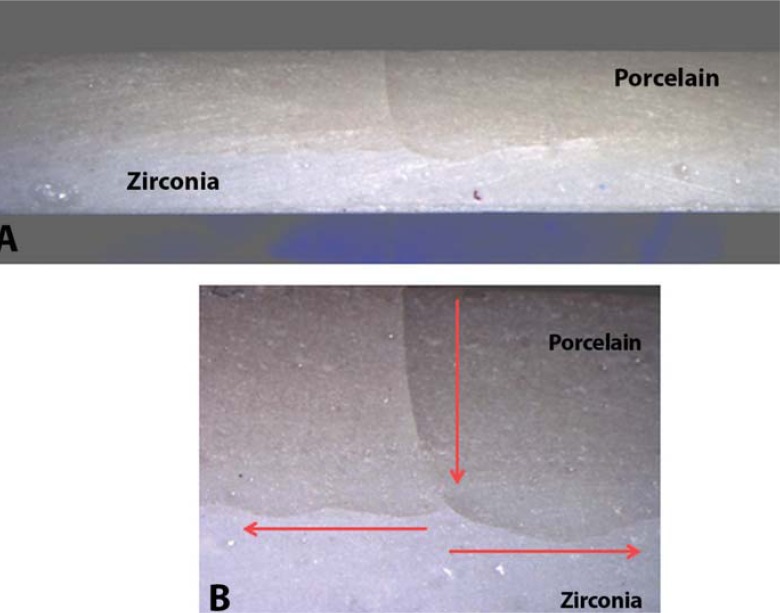
1-mm-thick porcelain bar. Porcelain cracking (A) (10x). Origin of the crack in the porcelain surface and propagation toward the interface, from center to ends (B) (40x)

**Figure 4:**
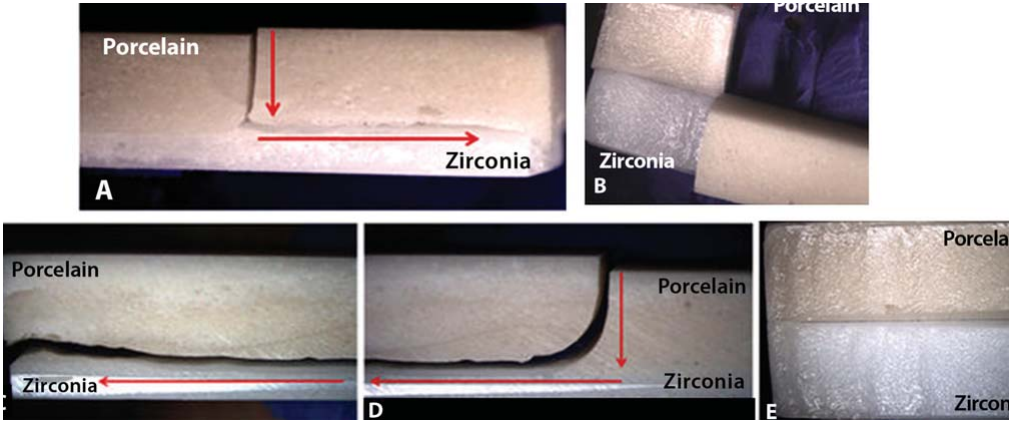
2-mm-thick (A; B) and 3-mm-thick (D; C; E) porcelain bar. Defect propagation from porcelain surface to interface (10x) and toward the bar ends (A; C; D). Porcelain was detached from zirconia, which was classified as catastrophic failure (B; E) (10x)

The effect of porcelain thickness on the bilayer zirconia system was statistically significant (*p*=0.001), according to One-way ANOVA. The ceramic bar with 1 mm porcelain had higher flexural strength. Tukey's test did not identify a statistically significant difference between the bilayers (Table 2). Cracking predominated among groups with 1 mm and 2 mm porcelain thickness, catastrophic failures were found in 50% of specimens with 3 mm veneering ceramics, and delamination/chipping was present in 20% of bars with 2 mm of porcelain. The origin of failures occurred at the porcelain surface with propagation at the interface between zirconia and porcelain in all groups. Failures ran from the center to the edges in all groups (Figures 3 and 4). The proportion of porcelain detached from zirconia in catastrophic failures was larger in the 3 mm than in the 2 mm group (Figure 4).

**Table 2:** Flexural strengths (MPa) and types of failures

Groups	Flexural strength means		Type of failure		p-value	Grouping**
	(SD)	Cracking	Delamination/ chipping	Catastrophic		
1 mm	51.5 (9.84)	10 (100%)	0 (0%)	0 (0%)	0.001	A
2 mm	35.5 (9.03)	7 (70%)	2 (20%)	1 (10%)		B
3 mm	35.8 (6.01)	5 (50%)	0 (0%)	5 (50%)		B

** Groups that share a letter were not statistically significantly different (Tukey's test; α = 0.05) for flexural strength data.

**Figure 5:**
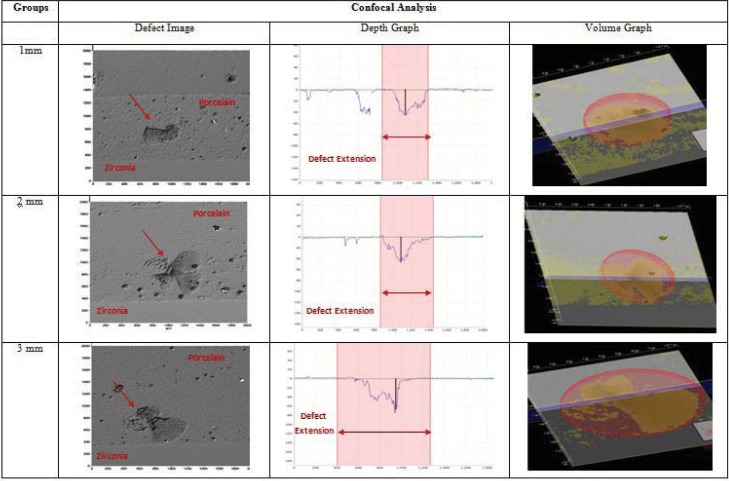
Representative images from confocal microscopy of the experimental groups: top profile, depth and volume extension of defects generated in the block specimens close to the interface

**Figure 6:**
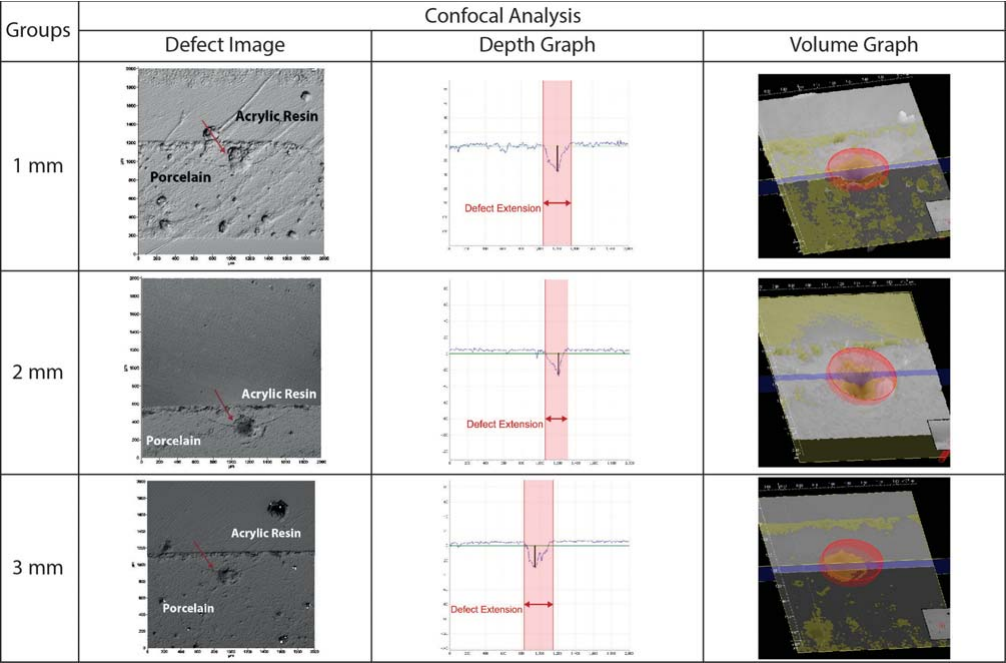
Representative images from confocal microscopy of the experimental groups: top profile, depth and volume extension of defects generated in the block specimens far from the interface

Average depth, extension, and volume of near and far indentations are shown in Table 3. Propagation of defects showed higher depth, extension, and volume close to zirconia, while all these parameters were lower when far from zirconia, except for depth in the 1 mm group. Figures 5 and 6 show defects generated in the block specimens.

**Table 3:** Mean value of depth, extension, and volume close to and far from zirconia

			Defect variables	
Indented region	Groups	Depth (μm)	Extension (μm)	Volume (μm^3^)
	1 mm	37.3	465.5	2.35
Close	2 mm	39.2	425.5	3.01
	3 mm	52.7	610.5	3.01
	1 mm	38	288.5	0.88
Far	2 mm	33.3	324.5	1.22
	3 mm	30.3	386	0.77

## Discussion

Investigating the failures in porcelain-zirconia infrastructures has become a major focus of research in the science of dental materials. In particular, understanding the role of residual stresses in these failures remains a challenge for researchers. In the present study, we showed that thin porcelain layers (1 mm) presented higher flexural strength and the indentation (defect) progression did not depend on porcelain thickness, but was more severe near the zirconia substrate than on the porcelain surface.

These results agree with Lima, et al.^15^ (2013) and White, et al.^25^ (2005), in which a thin veneer thickness provided higher flexural strength of bilayer ceramic specimens. These studies also presented cracking as the principal failure mode for low-thick bilayers.

However, in clinics^20^ ^,^ ^27^, the most frequent failure is cusp fractures of all-ceramic restorations because they present greater thicknesses of porcelain and are affected by residual and mechanical stresses^27^. The interface between porcelain and zirconia is sensitive to the poor heat transfer among materials^29^ and when thick layers of porcelain that have even lower thermal diffusivity are used, there is spalling of porcelain^27^.

Therefore, thickness of porcelain increased from 1 mm to 2 mm significantly increases the thermal transient stress gradients between the zirconia and veneer^29^, explaining thus the change in failure patterns between 1 mm and 2 mm groups, and justifying the occurrences of delamination/chipping. The residual stress scenario was even worse for the 3 mm specimens, also justifying the higher number of catastrophic (delamination) failures. The predominance of cracks in the 1 mm group can be explained by the compressive stresses due to the tempering effect^17^ that favored the mechanical strength of such bilayer system.

One can argue that bilayered disks are not the same as crowns. Nevertheless, the layered model, while simplistic in its geometry, provides a physical basis for investigating the role of material properties and the effect of thickness in the idealization of all-ceramic crowns^13^, besides facilitating the assessment of the origin and propagation of the defect^14^. With regard to residual stresses, the main difference between crowns and disks is a tendency of the latter for residual compressive stresses to increase with an increase in thickness from 1 to 2 mm^1^. However, the more aggressive damages seen after the indentation of 2 mm specimens did not confirm such observation, which is probably related to the fact that we used a slow cooling protocol that led to relaxation of stresses within the layers.

In the present study, we were not able to quantify spatially residual stresses in the porcelain layers using Vickers indentations, although this has been done before with the same method on curved surfaces^1^. Thus, we only observed the occurrence of crack propagation near the interface, confirming the presence of tensile stresses, as shown previously^4^ ^,^ ^8^ ^,^ ^10^ ^,^ ^13^ ^–^ ^17^ ^,^ ^27^. Most of the cracks extended latero-inwardly, confirming a tendency for porcelain chipping instead of porcelain debonding from zirconia. Therefore, tensile residual stresses occur predominantly parallel rather than perpendicular to the porcelain/zirconia interface^2^. Based on the damage extension, the cracks ran more rapidly in regions close than far from zirconia. Although the 1 mm group showed higher defect depths near the porcelain surface, crack length and volume remained relatively small. The higher depth in this group can be explained by the presence of a pore in the vicinity. The presence of such irregularity inside the layers is a limitation of using the indentation method, because the crack can easily interact with the pore in the bulk. On the other hand, compressive stresses are prevalent near the porcelain surface^5^ regardless of the veneer thickness^17^, as shown by similar defect sizes and crack lengths. However, this type of residual stress is more intense in fast cooled bilayers.

The use of Vickers indentations for measuring residual stress has already been proved itself useful^1^ ^,^ ^2^. Though it is not appropriate to compare the mechanical properties measured by this method, the results of the present study were consistent in showing that, depending on the thickness of the porcelain, residual stress may difficult (compressive stress) or favor (tensile stress) crack propagation. It is important to note that the application of a 10-kg load for 10 s, as in the Vickers indentation test, is not analogous to the masticatory load, but it is a way to promote the occurrence of a severe defect and to probe the fracture resistance of the porcelain. We are aware of the residual stresses imparted by this indentation method but, in the present study, it helped driving the crack along with the residual tensile stresses inside the porcelain.

Other techniques for the observation of cracks and defects are scanning electron microscopy (SEM), transillumination, and microtomography. The first method is important for surface assessment but does not allow in-depth observations. The second method allows us to detect internal defects, precluding these measurements. Finally, microtomography facilitates the observation of crack propagation but requires considerable specimen preparation, which can distort the orientation of cracks, leading to difficulty in damage assessment^1^. The use of confocal microscopy showed satisfactory results in the analysis of defects found in the ceramic bilayer system. It proved to be a quick and simple technique that does not require costly sample preparation and that offers crucial information on defects in the surface and beneath it, being a viable method for observing ceramic defects. Thus, we believe that the association of confocal microscopy and the Vickers indentation is an effective resource for observing defects/crack propagation and it highlights the presence of residual stresses in ceramic materials.

The anticipated hypotheses (H1 and H2) were accepted. Limitations of this study were the absence of realistic scenarios resembling the oral environment, such as the completion of the flexural test or thermalcycling in water, which were avoided to minimize the occurrence of subcritical crack growth. Further researches should be developed using confocal microscopy to assess the pathway of defects in complex geometries, such as crowns and bridges. In addition, it is important to evaluate the behavior of cracks or defects generated under fatigue loading or other types of damage^28^.

## Conclusions

The thinnest (1 mm) porcelain/zirconia specimens presented higher flexural strength. Defects in ceramics close to the porcelain/zirconia interface grew faster than defects close to the porcelain surface for all thicknesses, suggesting that the occurrence of residual tensile stresses is higher at this site.
